# Effectiveness of Reflex Integration Exercises on Sensory-Motor Outcomes in Children With Autism Spectrum Disorder: A Quasi-experimental Study

**DOI:** 10.7759/cureus.111970

**Published:** 2026-07-02

**Authors:** Marzia A Bijle, Mandar Malawade

**Affiliations:** 1 Department of Pediatric Neurosciences Physiotherapy, Krishna College of Physiotherapy, Krishna Vishwa Vidyapeeth (Deemed to be University), Karad, IND

**Keywords:** autism spectrum disorder, bot-2, neurodevelopmental rehabilitation, paediatric physiotherapy, primitive reflexes, reflex integration, sensory-motor outcomes, sensory processing

## Abstract

Background

Autism spectrum disorder (ASD) is a complex neurodevelopmental condition associated with impairments in sensory processing, motor coordination, and postural control. A prominent yet underappreciated contributor to these deficits is the persistence of primitive reflexes beyond the expected developmental timeline. Reflexes such as the asymmetrical tonic neck reflex (ATNR), symmetrical tonic neck reflex (STNR), and tonic labyrinthine reflex (TLR) are brainstem-mediated automatisms that, under typical neurodevelopment, integrate within the first year of life. In children with ASD, their persistence disrupts voluntary motor control, sensory modulation, postural stability, and behavioral regulation, thereby amplifying functional limitations across daily activities.

Objective

This study aimed to evaluate the effectiveness of a structured 12-week reflex integration exercise program on primitive reflex inhibition, motor proficiency, and sensory processing in children with ASD aged four to 10 years.

Methods

An experimental pre-post interventional design was employed with a sample of 27 children (mean age 6.69 ± 2.38 years; 74.1% male) diagnosed with mild to moderate ASD, recruited through convenience sampling from a single tertiary care pediatric physiotherapy setting. Participants underwent a structured 12-week reflex integration exercise program targeting the ATNR, STNR, and TLR, delivered across four sessions per week at progressive intensities through three distinct four-week phases. Outcome measures included the Bruininks-Oseretsky Test of Motor Proficiency, Second Edition (BOT-2) for motor outcomes; the Short Sensory Profile (SSP) encompassing Sensory and Behavior subscales for sensory outcomes; and the Sally Goddard Primitive Reflex Testing Protocol for reflex inhibition. Data were analyzed using paired samples t-tests, with Cohen’s d computed to quantify effect size. Statistical significance was set at p < 0.05.

Results

Post-intervention findings demonstrated statistically significant improvements across all outcome domains. Motor proficiency improved markedly, with BOT-2 scores increasing from a pre-intervention mean of 32.41 ± 5.38 to a post-intervention mean of 40.93 ± 4.50. Sensory processing difficulties decreased substantially, with SSP Sensory subscale scores reducing from 57.07 ± 6.29 to 44.67 ± 4.37 and SSP Behavior subscale scores declining from 82.37 ± 7.98 to 63.00 ± 7.50. Significant bilateral inhibition of retained reflexes was demonstrated for the ATNR, STNR, and TLR.

Conclusion

A structured 12-week reflex integration exercise program produced clinically meaningful and statistically significant improvements in motor proficiency, sensory processing, behavioral regulation, and primitive reflex inhibition in children with ASD aged four to 10 years. The large effect sizes observed across all outcome domains underscore the neurophysiological relevance of targeting retained primitive reflexes as a foundational mechanism for broader sensorimotor development in this population. These preliminary findings suggest that reflex integration exercises may hold promise as a therapeutic component in pediatric physiotherapy for children with ASD; however, randomized controlled trials with larger samples are warranted before definitive clinical recommendations can be made.

## Introduction

Autism spectrum disorder (ASD) represents one of the most extensively studied and heterogeneous neurodevelopmental conditions of contemporary pediatric medicine, characterized by persistent difficulties in social communication and interaction, as well as restricted and repetitive patterns of behavior, that lead to considerable variability in functional impairment across multiple domains of development [1]. ASD in India has an estimated prevalence of 0.15%-1% (1-10 per 1,000 children) [2]. Motor coordination impairments affect approximately 85%-90% of children with ASD, presenting as deficits in postural control, gait, bilateral coordination, motor planning, and fine motor skills. Additionally, 45%-95% exhibit sensory processing abnormalities, including hypersensitivity to tactile and auditory stimuli, sensory-seeking behaviors, and vestibular dysregulation, impacting learning, participation, and emotional regulation [3].

A particularly important and emerging contributor to sensorimotor dysfunction in ASD is the atypical retention of primitive reflexes; while these involuntary, survival-based movement patterns are essential during early infancy, they are expected to fade as the central nervous system matures [4]. In children with ASD, however, the persistence of these reflexes past their typical developmental window acts as a persistent neurodevelopmental hurdle, actively interfering with the emergence of mature, voluntary motor control and further compounding sensorimotor dysfunction [5]. Under typical neurodevelopment, these reflexes are progressively inhibited and integrated as cortical control matures, typically within the first 12 months of postnatal life, thereby facilitating the transition from automatic reflexive responses to purposeful, coordinated voluntary movement [6]. When early reflexes fail to integrate, their persistence disrupts voluntary motor control and postural stability; this neurological interference directly impacts sensory processing, attention, and behavioral regulation-key domains in the functional profile of children with ASD [7]. Studies have documented that up to 83.33% of reflex patterns are dysfunctional in children with ASD compared to neurotypical peers, suggesting that primitive reflex retention may represent a clinically significant and frequently observed indicator of neuromotor developmental delay in this population, though its role as a primary diagnostic or pathological marker requires further validation through controlled longitudinal research [8]. Among the most clinically significant retained reflexes in ASD are the asymmetrical tonic neck reflex (ATNR), symmetrical tonic neck reflex (STNR), and tonic labyrinthine reflex (TLR). The retention of ATNR causes involuntary limb extension on the side toward which the head rotates, impairing bilateral coordination and visual-motor integration [9]. When STNR is unintegrated, it generates conflicting extensor and flexor responses during head movement in quadruped positions, which interfere with crawling, cross-lateral movement, and fine motor control, while the TLR, when persistent, causes disruption of postural tone and gravitational orientation through labyrinthine mismatch, leading to impaired balance, spatial awareness, and proprioceptive processing [10]. In children with ASD, the cumulative neuromotor load of these unintegrated reflexes includes emotional dysregulation, altered arousal control, and attentional difficulties-domains that are separately identified as key components of the ASD phenotype [11].

Reflex integration exercises are theoretically grounded in the principle of neuroplasticity; it has been proposed that repetitive activation of retained primitive reflexes, combined with intentional inhibition techniques, may harness neuroplastic mechanisms to encourage maturation of descending cortical inhibitory pathways, potentially facilitating integration of reflex patterns that persist beyond typical developmental windows and thereby improving sensorimotor regulation and functional outcomes [12]. It has further been hypothesized that such interventions may promote synaptogenesis and strengthen corticospinal tract projections that override brainstem-mediated reflex activity through controlled movement sequences requiring cortical suppression of primitive reflex responses, though direct neurophysiological evidence confirming these mechanisms in children with ASD remains limited [13]. A growing body of evidence has demonstrated that targeted reflex integration programs yield significant improvements in motor coordination, postural control, balance, sensory processing, behavioral regulation, and attention in children with neurodevelopmental disorders [14].

Nevertheless, rigorous experimental data specifically evaluating the isolated effects of structured reflex integration exercises on standardized sensorimotor outcomes in children with ASD remain notably sparse, as the majority of existing research either embeds such protocols within broader multimodal interventions or directs its focus toward other neurodevelopmental populations, thereby leaving a substantive gap in the literature regarding the targeted therapeutic impact of these approaches in this specific clinical population [15]. The present study, therefore, addresses a clearly identified gap in the existing evidence base by examining the effectiveness of a structured, progressive reflex integration exercise program specifically targeting the ATNR, STNR, and TLR on validated sensorimotor outcomes in children with ASD.

## Materials and methods

This was a single-group quasi-experimental pre-post interventional study conducted at the Department of Pediatric Physiotherapy, Krishna College of Physiotherapy, Krishna Vishwa Vidyapeeth (Deemed to be University), Karad, Maharashtra, India. Ethical clearance was obtained from the Institutional Ethics Committee of Krishna Vishwa Vidyapeeth (Approval No. KVV/IEC/10/2025). Written informed consent was obtained from parents or legal guardians of all participants prior to study enrollment, and written assent was additionally obtained from children aged four years and above. The study was conducted in strict accordance with the ethical principles of the Declaration of Helsinki.

The study was conducted from December 2025 to April 2026. Participant recruitment and baseline assessments were initiated in December 2025. The 12-week reflex integration exercise intervention was administered from January 2026 to March 2026, and post-intervention outcome assessments were completed in April 2026.

The sample size was estimated a priori based on a pilot estimate of the primary outcome variable (Bruininks-Oseretsky Test of Motor Proficiency, Second Edition (BOT-2) scores), using the formula for paired samples: $n=\left( Z_{\alpha/2}+Z_{\beta} \right)^{2}\times 2\sigma^{2}/d^{2}$, where a two-tailed significance level of *α* = 0.05 (*Z* = 1.96), a power of 80% (*Z_β_* = 0.84), an expected mean difference of 8 points, and a standard deviation of 10 points were assumed based on previously published sensorimotor intervention studies in children with ASD, yielding a minimum required sample of 25 participants [16]. To account for potential attrition, 27 participants were enrolled. It is acknowledged as a limitation of this study that the original sample size estimation was based on a prevalence formula rather than an intervention effect size calculation; the above post hoc power verification confirms that the enrolled sample was nonetheless adequately powered to detect the observed effects. Consecutive convenience sampling was employed, and 27 children meeting the predetermined eligibility criteria were enrolled. All 27 enrolled participants completed the full 12-week intervention and post-intervention assessment without dropout or loss to follow-up, yielding a retention rate of 100%. No adverse events or intervention-related complications were reported during the study period. The study was conducted over a period of 12 weeks. The intervention was administered four times per week, totaling 48 sessions.

Inclusion criteria comprised children aged 4-10 years; a confirmed clinical diagnosis of mild to moderate ASD (Diagnostic and Statistical Manual of Mental Disorders, 5th Edition (DSM-5) severity levels 1 and 2, corresponding to requiring support and requiring substantial support, respectively) by a pediatric neurologist or developmental pediatrician, as documented in the child's medical records at the time of enrolment; documented presence of retained ATNR, STNR, and/or TLR as determined by the Sally Goddard Reflex Testing Protocol at baseline assessment; and sufficient capacity to follow simple verbal and demonstrated instructions to participate in structured therapeutic activities [17,18].

Children were excluded if they had coexisting neurological conditions such as cerebral palsy, Down syndrome, or severe intellectual disability in addition to ASD; visual, auditory, or speech impairments of sufficient severity to preclude valid assessment or therapeutic participation; or significant musculoskeletal or cardiorespiratory conditions limiting physical activity or motor performance assessment.

Outcome measures

Three validated instruments were administered at baseline (Week 0) and immediately following completion of the 12-week intervention program (Week 12) by trained assessors who were not involved in delivering the intervention. It is acknowledged, however, that given the single-group pre-post design of this study, complete assessor blinding to the temporal status of assessments (baseline versus post-intervention) was not feasible. Assessors were blinded to the specific therapeutic objectives and exercise content of individual intervention sessions, though this does not preclude awareness of the assessment timepoint. This represents an inherent methodological limitation of the study design and has been noted accordingly in the Limitations section.

Motor Proficiency: BOT-2

The BOT-2 Short Form was employed to assess overall motor proficiency across domains of bilateral coordination, balance, and upper-limb coordination-subcomponents directly influenced by the retained reflexes targeted in the intervention. The BOT-2 is standardized for use in children aged 4-21 years and demonstrates high test-retest reliability (r > 0.80), strong construct validity, and clinical sensitivity to motor change in children with neurodevelopmental disorders.

Sensory Processing: Short Sensory Profile 2

The Short Sensory Profile 2 (SSP2), a standardized caregiver-completed questionnaire evaluating sensory processing patterns, was administered to assess changes in sensory modulation across tactile, auditory, visual, vestibular, and proprioceptive domains [19]. The tool encompasses a Sensory subscale (maximum score 70) and a Behavior subscale (maximum score 100), wherein lower post-intervention scores indicate reduced sensory processing difficulties and behavioral dysregulation, respectively. The SSP2 demonstrates high internal consistency (Cronbach’s α > 0.80) and strong validity for use in children with ASD.

Primitive Reflex Assessment: Sally Goddard Reflex Testing Protocol

Primitive reflex retention was assessed using the validated 5-point ordinal rating scale devised by Goddard, independently for the ATNR (right and left), STNR (flexion and extension), and TLR (flexion and extension). Scores range from 0 (absence of reflex activity) to 4 (full, uncontrolled reflex response), with higher scores reflecting greater degrees of reflex retention and corresponding neuromotor immaturity.

Intervention protocol

The reflex integration exercise program was delivered over 12 consecutive weeks, comprising four supervised sessions per week at the pediatric physiotherapy department, with each session lasting between 30 and 45 minutes, inclusive of rest intervals. The intervention was structured across three progressive four-week phases (Phase I: Weeks 1-4; Phase II: Weeks 5-8; Phase III: Weeks 9-12), with escalating intensity from mild to moderate across phases to ensure progressive neuromotor challenge in accordance with the principles of motor learning and neuroplasticity-driven rehabilitation. Three distinct exercise streams were implemented, each targeting a specific retained primitive reflex pattern, as shown in Table 1. The exercise protocol was developed de novo by the authors based on theoretical principles of primitive reflex inhibition and neuroplasticity-driven rehabilitation derived from the existing literature [12,13,20]. As no single standardized reflex integration exercise protocol for children with ASD currently exists in the published literature, the present protocol represents an original structured intervention, and its replication and validation in future trials are encouraged.

**Table 1:** Phase-Wise Progression of Reflex Integration Exercises for Primitive Reflexes (ATNR, STNR, and TLR) Over 12 Weeks ATNR: asymmetrical tonic neck reflex; STNR: symmetrical tonic neck reflex; TLR: tonic labyrinthine reflex

Exercises	Phase I (Weeks 1-4)	Phase II (Weeks 5-8)	Phase III (Weeks 9-12)	Intensity
ATNR				
Cross crawl	3 sets × 10 reps	3 sets × 15 reps	3 sets × 20 reps	Mild → moderate
Lizard crawl	Hold 3 min each side	Hold 4 min each side	Hold 5 min each side	Mild → moderate
Sword march	3 sets × 8 reps each side	3 sets × 10 reps each side	3 sets × 12-15 reps each side	Mild → moderate
Half kneeling catches	Maintain 2 min	Maintain 3 min	Maintain 4-5 min	Mild → moderate
Eye tracking with head rotation	1 set × 30 sec each side	1 set × 45 sec each side	1 set × 1 min each side	Mild → moderate
Bear walk	1 set × 2 min	1 set × 3 min	1 set × 4 min	Mild → moderate
STNR				
Cat-camel stretch (quadruped)	3 sets × 7 reps	3 sets × 10 reps	3 sets × 12-15 reps	Mild → moderate
Cross crawling	1 set × 2 min	1 set × 3 min	1 set × 4 min	Mild → moderate
Quadruped reach-outs	3 sets × 10 reps	3 sets × 12 reps	3 sets × 15 reps	Mild → moderate
Rocking horse	3 sets × 2 min each side	3 sets × 3 min each side	3 sets × 4 min each side	Mild → moderate
TLR				
Superman pose	Hold 2 min	Hold 3 min	Hold 4 min	Mild → moderate
Knee-to-chest with side-to-side roll	3 sets × 2 reps each side	3 sets × 3 reps each side	3 sets × 4 reps each side	Mild → moderate
Rolling like a log	20 sec	30 sec	40 sec	Mild → moderate
Superman ball push	As tolerated	Increase time gradually	Maximum tolerated duration	Mild → moderate

While all 27 participants completed the full 12-week protocol, the implementation of reflex integration exercises in children with ASD was not without challenges. In the initial sessions of Phase I, several children demonstrated difficulty sustaining attention for the required duration of exercises, particularly during static holds such as the lizard crawl and superman pose. Sensory sensitivities, including tactile defensiveness and vestibular hypersensitivity, posed initial barriers for a subset of participants during floor-based and rolling exercises such as the knee-to-chest with side-to-side roll and rolling like a log. Behavioral dysregulation in the form of resistance to positional changes and difficulty following sequential instructions was observed in some children, particularly those with higher scores on the SSP Behavior subscale at baseline. These challenges were addressed through gradual desensitization, use of visual schedules and demonstrated modeling, incorporation of rest intervals, and consistent caregiver presence during sessions to provide reassurance. Exercise durations and repetitions were initially adjusted within the prescribed range where necessary to accommodate individual tolerance, with progressive increments applied as children habituated to the therapeutic environment. No participant required permanent modification or exclusion of any exercise from their program.

Statistical analysis

All statistical analyses were performed using IBM SPSS Statistics software, Version 25.0 (IBM Corp., Armonk, NY, USA). Descriptive statistics, comprising means and standard deviations, were calculated for all continuous variables at baseline and post-intervention. Normality of distribution for all outcome variables was confirmed using the Shapiro-Wilk test (all p > 0.05), validating the use of parametric inferential analysis. Within-group pre-post comparisons for all outcome measures were performed using the paired samples t-test. Effect sizes were quantified using Cohen’s d, with values ≥ 0.80, ≥0.50, and ≥0.20, respectively, interpreted as large, medium, and small effects in accordance with established conventions. The level of statistical significance was set at p < 0.05.

## Results

A total of 27 children with ASD were included, with an age range of 4-10 years. The sample showed male predominance with 20 males (74.1%) and seven females (25.9%).

The analysis of BOT-2 demonstrated a statistically significant improvement following the 12-week reflex integration program, as shown in Table 2. The mean score increased from 32.41 ± 5.38 at baseline to 40.93 ± 4.50 post-intervention, with a mean difference of +8.52 points (95% CI: 7.16-9.88; t = 12.887; p < 0.001; Cohen’s d = 2.48), indicating clinically meaningful improvement in overall motor proficiency.

**Table 2:** Comparison of Pre- and Post-intervention BOT-2 Scores SD: standard deviation; BOT-2: Bruininks-Oseretsky Test of Motor Proficiency, Second Edition

Outcome measure	Pre mean ± SD	Post mean ± SD	Mean Diff	t-value	p-value	Cohen’s d
BOT-2 score (/88)	32.41 ± 5.38	40.93 ± 4.50	+8.52	12.887	<0.001	2.48

Analysis of the SSP, as shown in Table 3, revealed statistically significant improvements across both subscales following the reflex integration program. The Sensory subscale showed a mean reduction of 12.41 points (pre: 57.07 ± 6.29; post: 44.67 ± 4.37; 95% CI: −14.49 to −10.32; t = 12.230; p < 0.001; Cohen’s d = 2.35), indicating reduced sensory processing difficulties. Similarly, the Behavior subscale decreased by 19.37 points (pre: 82.37 ± 7.98; post: 63.00 ± 7.50; 95% CI: −22.56 to −16.18; t = 12.483; p < 0.001; Cohen’s d = 2.40), reflecting reduced sensory-related behavioral dysregulation. As lower SSP scores denote reduced symptom severity, these findings indicate clinically meaningful improvements across sensory and behavioral domains, supported by large effect sizes (d > 2.30).

**Table 3:** Comparison of Pre- and Post-intervention SSP Scores SSP: Short Sensory Profile; SD: standard deviation

SSP subscale	Pre mean ± SD	Post mean ± SD	Mean Diff	t-value	p-value	Cohen’s d
Sensory (/70)	57.07 ± 6.29	44.67 ± 4.37	−12.41	12.230	<0.001	2.35
Behavior (/100)	82.37 ± 7.98	63.00 ± 7.50	−19.37	12.483	<0.001	2.40

Table 4 demonstrates the assessment of primitive reflexes measured using a 5-point rating scale suggested by Goddard. A significant reduction in all three reflexes was observed following the intervention.

**Table 4:** Primitive Reflex Assessment Pre- and Post-intervention Comparison All p-values indicate extreme statistical significance. ATNR: asymmetrical tonic neck reflex; STNR: symmetrical tonic neck reflex; TLR: tonic labyrinthine reflex; SD: standard deviation

Reflex	Position/side	Pre mean (SD)	Post mean (SD)	t-value	p-value	Cohen’s d
ATNR	Right	2.89 (0.75)	1.22 (0.70)	12.748	<0.001	2.30
ATNR	Left	2.78 (0.85)	1.15 (0.66)	8.760	<0.001	2.16
STNR	Flexion	2.89 (0.75)	1.33 (0.73)	10.096	<0.001	2.11
STNR	Extension	2.96 (0.71)	1.78 (0.75)	9.894	<0.001	1.62
TLR	Flexion	2.04 (0.71)	0.89 (0.70)	11.177	<0.001	1.63
TLR	Extension	2.52 (0.89)	1.52 (0.64)	7.080	<0.001	1.31

The findings revealed statistically significant improvements across all assessed domains: motor proficiency, sensory processing, behavioral regulation, and primitive reflex inhibition. The observed mean improvement in BOT-2 scores of 8.52 points exceeds the minimal clinically important difference (MCID) reported for motor proficiency measures in children with neurodevelopmental disorders, and the large Cohen's d effect sizes (ranging from 1.31 to 2.48 across all outcome measures) provide quantitative support for the practical magnitude of these changes. It is acknowledged, however, that formal pre-specified MCID thresholds for the BOT-2 Short Form and SSP2 in children with ASD have not been definitively established in the literature, and the clinical interpretation of these findings should therefore be considered in light of this limitation.

## Discussion

A clinically significant improvement was observed in the bilateral coordination, balance, and upper-limb coordination domains, evaluated as primary subcomponents of the BOT-2 in this study and directly linked to the neuromotor sequelae of primitive reflex retention. These results are consistent with the growing body of evidence supporting structured movement-based interventions for motor improvement in ASD. Alahmari et al. demonstrated that sensory-motor integration exercises, as an evidence-based rehabilitative technique, produced significant improvements in motor performance in children with ASD [21]. Savarese et al. similarly reported substantively. It is speculated, though not empirically confirmed within the present study design, that the neuromotor specificity of the reflex integration approach targeting the proposed underlying mechanism of voluntary motor inhibition rather than surface-level motor performance may have contributed to the observed improvements [22]. As this study employed a single-group pre-post design without a comparator condition, no causal inferences regarding the mechanism of action can be drawn, and this interpretation remains theoretical and hypothesis-generating in nature. This mechanistic interpretation is substantiated by Melillo et al., who suggested that retained primitive reflexes constitute a fundamental neurological challenge to voluntary motor development in ASD via impairing the descending corticospinal inhibitory control, and that controlled exercise that specifically inhibits them can promote synaptogenesis and improve neuroplasticity in higher cortical motor areas [20]. Acuña et al. conducted a systematic review of randomized controlled trials on Ayres Sensory Integration for children aged 0-12 years, confirming that when delivered with fidelity, sensory integration-based interventions produce significant improvements in individualized occupational performance goals, function, and participation in autistic children [23]. The 12-week protocol used in this study has a progressive, phase-based structure that increases in intensity from mild in Phase I to moderate in Phase III. This structure reflects established principles of progressive motor challenge in neuroplasticity-driven rehabilitation and is consistent with research showing that intense, increasingly demanding motor training promotes descending inhibitory control and neural pathway consolidation. Hirose et al. clearly supported the current findings, reporting significant reductions in ATNR retention accompanied by significant improvements in fine motor coordination following a 12-week exercise intervention in children with ASD and ADHD [1].

The foundational clinical work of Tomchek and Dunn, who found that 95% of children with ASD exhibit measurable sensory processing dysfunction with notable abnormalities, particularly in tactile sensitivity, auditory filtering, and sensory-seeking behavior, contextualizes these findings [24]. Consoli et al. emphasized that sensory processing difficulties in ASD are important to the clinical presentation of the condition, associated with distinct patterns of social communication, repetitive behaviors, and comorbidities [25]. Camino-Alarcón et al.'s comprehensive review verified that sensory integration-based therapies significantly enhance children with ASD's sensory processing results, especially in the proprioceptive, vestibular, and tactile domains [26]. Oh et al., through meta-analysis, further confirmed the clinical effectiveness of sensory integration therapy in improving sensory processing outcomes and daily functional performance across pediatric populations through meta-analytic synthesis [27].

Beyond isolated sensory improvements, the study's considerable increase in SSP Behavior subscale scores suggests a similar beneficial effect on the behavioral aspect of sensory modulation. Children with ASD showed improvements in behavioral regulation, emotional stability, and adaptive responses after reflex integration therapy, according to Masgutova et al., whose Masgutova Neurosensorimotor Reflex Integration (MNRI) program shares theoretical and mechanistic underpinnings with the current intervention [10]. The vestibular and proprioceptive stimulation inherent in the reflex integration exercises employed in this study, particularly the TLR-targeting exercises involving gravitational orientation challenges, likely played a specific role in driving these sensory improvements. Vestibular-targeted interventions are a clinically significant therapeutic avenue in ASD management, according to Chisari et al., who showed that vestibular function is severely impaired in children with ASD, with direct implications for postural control and sensory integration [28]. Vestibular and proprioceptive exercises significantly reduce sensory-seeking behaviors and enhance postural stability in children with ASD, according to Hariri et al. [29].

The finding of statistically significant inhibition of all three targeted primitive reflexes across all assessed positions and sides represents the central and distinguishing contribution of the present study. The study is supported by Melillo et al., who demonstrated that retained primitive reflexes in ASD are associated with abnormal interregional brain connectivity and maturational delays within corticospinal tract development; it has been theoretically proposed that reflex integration exercises may stimulate neuroplasticity and strengthen descending inhibitory control over brainstem-mediated reflex activity [22], though the present study did not incorporate neurophysiological or neuroimaging measures and therefore cannot confirm or demonstrate this mechanism directly [20]. Additionally, Melillo et al. found strong correlations between hemisphere connectivity asymmetry and maintained basic reflexes in ASD, offering neurobiological proof that reflex integration might promote beneficial brain remodeling in this group [13].

It has been hypothesized in the literature that reflex inhibition through structured exercise may operate by gradually strengthening descending corticospinal pathways, theoretically allowing higher cortical centers to override brainstem-mediated reflex activity through repeated practice of movement patterns requiring simultaneous reflex activation and voluntary cortical suppression [20]. While the behavioral and motor outcomes observed in the present study are consistent with this theoretical framework, they do not constitute direct neurophysiological evidence of these mechanisms, and caution is warranted in interpreting the findings as confirmation of the proposed neurobiological pathway [20]. This mechanism is similar to the normal developmental trajectory of reflex integration, which is disturbed in ASD. By showing that primitive reflex persistence in preschoolers is strongly linked to deficiencies in gross motor skills, balance, and coordination, Gieysztor et al. established the fundamental neuromotor rationale for focusing on reflex inhibition in pediatric rehabilitation [30].

Collectively, the findings of the present study underscore the therapeutic value of a structured primitive reflex integration protocol as a neuromotor intervention for children with ASD. By addressing the foundational neurological mechanisms underlying voluntary motor dysfunction rather than targeting surface-level behavioral symptoms, this approach yielded meaningful and measurable gains across motor coordination, balance, sensory processing, and behavioral regulation domains. The progressive, phase-based design of the 12-week protocol may have provided the neuromotor conditions theoretically conducive to gradual cortical maturation and descending inhibitory pathway consolidation, as proposed in the neuroplasticity literature upon which the intervention was rationally constructed [12,13,20]; however, as neither cortical maturation nor pathway consolidation were directly measured in this study, this interpretation remains inferential and cannot be considered a demonstrated outcome of the present investigation. These outcomes collectively suggest that primitive reflex retention represents a modifiable neuromotor target in ASD rehabilitation, and that its systematic inhibition through controlled movement training may serve as a clinically viable adjunct to existing therapeutic frameworks. Future investigations employing larger sample sizes, active control conditions, and extended follow-up periods are warranted to further delineate the long-term neuromotor and behavioral benefits of reflex integration therapy and to establish standardized dosage parameters for its broader clinical application.

Limitations

This study has a few limitations that should be considered when interpreting the findings. The absence of a control group makes it difficult to attribute the observed improvements solely to the intervention, as natural developmental changes may have contributed to the outcomes. The small sample size of 27 children from a single center limits how broadly these results can be applied to other pediatric ASD populations. Recruitment through convenience sampling may have introduced selection bias, and the absence of dropouts, while favorable for statistical completeness, may also reflect the highly supervised single-center nature of the study and the convenience sampling approach employed, and may not be representative of retention rates expected in larger or more diverse clinical settings. Assessor blinding was incomplete; while assessors were not involved in delivering the intervention and were unaware of session-specific therapeutic content, complete blinding to the assessment timepoint (baseline versus post-intervention) was not achievable within the single-group pre-post design employed. This introduces a potential risk of performance or expectation bias in outcome measurement, and future studies should incorporate independent blinded assessors operating under stricter blinding protocols. Since follow-up was restricted to 12 weeks, it remains unclear whether the sensorimotor gains achieved were maintained over time. The behavioral and sensory challenges encountered during exercise delivery, including attentional difficulties, tactile defensiveness, and resistance to positional changes, highlight the need for individualized adaptation strategies when implementing reflex integration protocols in clinical settings and underscore the importance of caregiver involvement and structured environmental supports. Future studies with randomized controlled designs, larger samples, and longer follow-up periods would help confirm and build upon the findings reported here.

## Conclusions

This pre-post interventional study demonstrated that a structured 12-week reflex integration exercise program produced statistically significant and clinically meaningful improvements across all sensorimotor outcome domains in children with ASD aged four to 10 years. Motor proficiency assessed by the BOT-2 improved substantially, reflecting gains in bilateral coordination, balance, and upper-limb function, while sensory processing difficulties and behavioral dysregulation measured by the SSP diminished markedly following intervention. Targeted inhibition of all three retained primitive reflexes-ATNR, STNR, and TLR-was confirmed across all assessed positions, with large effect sizes indicating a substantial magnitude of change across all behavioral and functional outcome domains. While these findings are theoretically consistent with neuromotor reorganization involving descending corticospinal inhibitory pathways as proposed in the literature, neither cortical maturation nor corticospinal pathway strengthening was directly measured in this study, and such neurophysiological conclusions cannot be drawn from behavioral outcome measures alone. These preliminary findings suggest that reflex integration may represent a clinically promising therapeutic component within pediatric physiotherapy for children with ASD, though the designation of this approach as neurophysiologically grounded awaits confirmation through future studies incorporating neuroimaging and electrophysiological outcome measures alongside randomized controlled designs. Still, the quasi-experimental single-group design of this study precludes definitive causal inference. Randomized controlled trials incorporating active control conditions, larger and more diverse samples, neuroimaging measures, and extended follow-up periods are necessary before these findings can inform evidence-based clinical practice guidelines.
